# Integrating UAV visible and multispectral imagery to assess grazing-induced vegetation responses in sandy grasslands

**DOI:** 10.3389/fpls.2025.1730583

**Published:** 2025-12-11

**Authors:** Qiang Guan, Mingyang Jiang, Wen Du, Xueyan Chen, Baolong Yan

**Affiliations:** 1 College of Computer Science and Technology, Inner Mongolia Minzu University, Tongliao, China; 2 College of Information and Electrical Engineering, Shenyang Agricultural University, Shenyang, China; 3 College of Engineering, Inner Mongolia Minzu University, Tongliao, China; 4 Innovation Center for Intelligent Forage Equipment, Inner Mongolia Minzu University, Tongliao, China; 5 College of Grassland Science, Inner Mongolia Minzu University, Tongliao, China

**Keywords:** sandy grasslands, multi-source remote sensing, unmanned aerial vehicle, grazing intensity, spectral indices, feature selection

## Abstract

**Introduction:**

Monitoring grazing intensity is crucial for maintaining ecological balance and promoting the sustainable management of sandy grasslands. Traditional ground surveys and single-source remote sensing often lack the spatial resolution, spectral richness, and robustness required to accurately characterize heterogeneous grazing impacts. Unmanned aerial vehicle (UAV)-based multi-source remote sensing provides fine-scale, repeatable observations that can overcome the limitations of traditional field surveys.

**Methods:**

Grazing experiments were conducted in the sandy grasslands of Inner Mongolia, China, using UAVs to capture visible and multispectral imagery across plots subjected to different grazing intensities. Spectral responses were analyzed using mean–variance statistics and Tukey’s multiple comparison tests. A series of novel spectral indices were constructed based on separability analysis and integrated with traditional vegetation indices to address the limited sensitivity of conventional indices and multi-index feature redundancy. An automatic incremental feature selection (AIFS) algorithm was developed to adaptively optimize the feature subset and enhance model robustness, with a support vector machine classifier, k-nearest neighbor, and random forest used for grazing intensity recognition.

**Results:**

Distinct spectral responses to grazing disturbance were observed: visible bands increased with grazing intensity due to enhanced soil background effects, while red-edge and near-infrared bands effectively captured reductions in chlorophyll content and canopy structure under moderate to severe grazing. Traditional vegetation indices were sensitive to extreme grazing, whereas the proposed indices showed superior performance in distinguishing moderate grazing levels. The AIFS-optimized feature subset reduced redundancy and improved model accuracy, achieving the highest recognition performance (OA=92.13%, Kappa=88.99%)—outperforming models using all features or single-source data.

**Discussion:**

Integrating UAV visible and multispectral imagery with intelligent feature selection enhances the detection of grazing-induced vegetation responses. This approach provides a robust framework for high-precision grassland monitoring and sustainable ecological management in arid and semi-arid regions.

## 1 Introduction

Grasslands are globally significant ecosystems, providing essential resources for livestock production and playing a vital role in maintaining ecological balance, sequestering carbon, and conserving water (Zhao et al., 2020). China has extensive grasslands, with the sandy grasslands of Inner Mongolia serving as a typical example, which function in both ecological protection and grazing production. However, long-term overgrazing and other human disturbances have caused severe degradation of sandy grasslands, leading to vegetation loss, wind erosion, and biodiversity decline (Lin et al., 2022; Jiang et al., 2024). Grazing intensity is a key indicator of grassland exploitation and degradation, and its accurate monitoring is vital for the ecological protection and sustainable use of sandy grasslands. Therefore, there is an urgent need to develop efficient and precise monitoring methods to support scientific management and policy formulation (Reinermann et al., 2020; Ali and Kaul, 2025).

Currently, monitoring grazing intensity on grasslands mainly relies on traditional field surveys and sampling plots. These methods remain direct and reliable, but are time-consuming, labor-intensive, and limited in spatial coverage, making dynamic monitoring challenging (Wang et al., 2022; Yu et al., 2025). Satellite remote sensing has been widely used for large-scale grassland monitoring; however, its spatial and temporal resolution often cannot meet the requirements for small-scale, detailed detection of grazing intensity in sandy grasslands (Ali et al., 2016; Wang et al., 2023). In contrast, unmanned aerial vehicle (UAV) remote sensing offers advantages such as flexibility, high resolution, and timely data acquisition, making it particularly suitable for detailed studies of sandy grassland surface characteristics and grazing disturbances (Lyu et al., 2022; Bazzo et al., 2023; Lyu et al., 2024). UAV-based studies typically acquire visible, multispectral, or hyperspectral imagery. Previous studies have demonstrated that hyperspectral data can capture subtle spectral variations, leading to improved accuracy in vegetation classification, degradation detection, and biomass estimation. However, its high equipment costs and complex data processing limit its large-scale application (Fernández-Habas et al., 2022; Wengert et al., 2022; Liu et al., 2023; Zhu et al., 2023; Zhang et al., 2024a). Therefore, cost-effective visible and multispectral data have become increasingly crucial for monitoring sandy grasslands (Rossi et al., 2019).

In recent years, visible-light imagery has been utilized to estimate grassland coverage and degradation levels due to its ease of acquisition and simplicity of processing (Possoch et al., 2016; Lussem et al., 2017, 2018; Zhang et al., 2022). Multispectral imagery, due to its rich spectral information, has been widely applied for estimating grass biomass and monitoring grazing disturbances (Orlando et al., 2022; Schucknecht et al., 2022; Gao et al., 2024; Zwick et al., 2024). However, monitoring based on a single data source often suffers from limited robustness across different grazing conditions, low generalizability, and inconsistent performance in distinguishing moderate grazing levels—one of the most critical yet difficult categories to detect. To address these limitations, multi-source data fusion has gradually emerged as a popular research focus (Barber et al., 2021; Liu et al., 2025). For example, some researchers extract key indicators, such as vegetation indices, and perform multi-indicator analyses to identify Zoysia japonica, thereby evaluating its cold tolerance (Ku et al., 2023). Other studies have developed multiple linear regression (MLR) and generalized additive models (GAM) to estimate grassland aboveground biomass (AGB) using texture features from UAV RGB imagery and vegetation indices from multispectral imagery (Pan et al., 2024). Researchers collected UAV RGB and multispectral imagery, combined vegetation structure variables with spectral features, and applied machine learning algorithms for image segmentation and species classification (Pranga et al., 2024). However, despite progress in multi-source fusion, existing studies still exhibit three major gaps: (1) fusion is often simple (e.g., direct concatenation of indices or original bands) and lacks targeted design based on spectral separability; (2) redundant or irrelevant features in multi-source datasets reduce model stability and generalizability; (3) limited attention has been given to constructing spectral indices specifically sensitive to grazing-induced vegetation responses, especially at moderate grazing intensities.

To address these challenges, this study focuses on detecting grazing intensity in a typical sandy grassland. By integrating visible and multispectral UAV data, the study examines the correlations and separability of spectral features. We construct targeted spectral indices (SIs) based on the physical mechanisms of vegetation response to grazing and fuse them with traditional vegetation indices. Concurrently, an automatic incremental feature selection (AIFS) method is applied to select multi-source features, reducing redundancy and enhancing adaptability to heterogeneous grazing conditions. Ultimately, by integrating and optimizing multiple-source SIs, the study achieves efficient and precise detection of grazing intensity in sandy grasslands, providing a novel approach for ecological monitoring and grassland management.

## 2 Materials and methods

### 2.1 Overview of the experiment site

The study was conducted at Manghatu Village, Gerchaolu Sumu, Zhalute Banner, Tongliao City, Inner Mongolia, China (44°62′24″N, 120°45′14″E) at an altitude of 482 m. The region features a temperate semi-arid continental climate with distinct seasons: dry, windy springs; hot summers with scarce rainfall; cool autumns with significant diurnal temperature variations; and long, cold winters. The climatic parameters described in this section were obtained from the China Meteorological Data Service Center and represent long-term averages based on a 30-year period (1995–2024). The long-term mean temperature is 5.5 °C, and the annual accumulated temperature ranges from 2400 to 2600 °C. Annual sunshine averages approximately 3000 hours, with a frost-free period of 115–130 days. Annual rainfall ranges from 300 to 400 mm, with the majority occurring between June and August, accounting for ~70% of the total precipitation. Annual evaporation exceeds 1800 mm, with an average relative humidity of 49%. The grassland is classified as a temperate mountain grassland, characterized by sandy soil with a pH of 7.6. The dominant species is *Agropyron cristatum*, with key associated species including *Lespedeza davurica*, *Cleistogenes squarrosa*, and *Carex duriuscula*.

### 2.2 Experimental design

**Figure 1:**
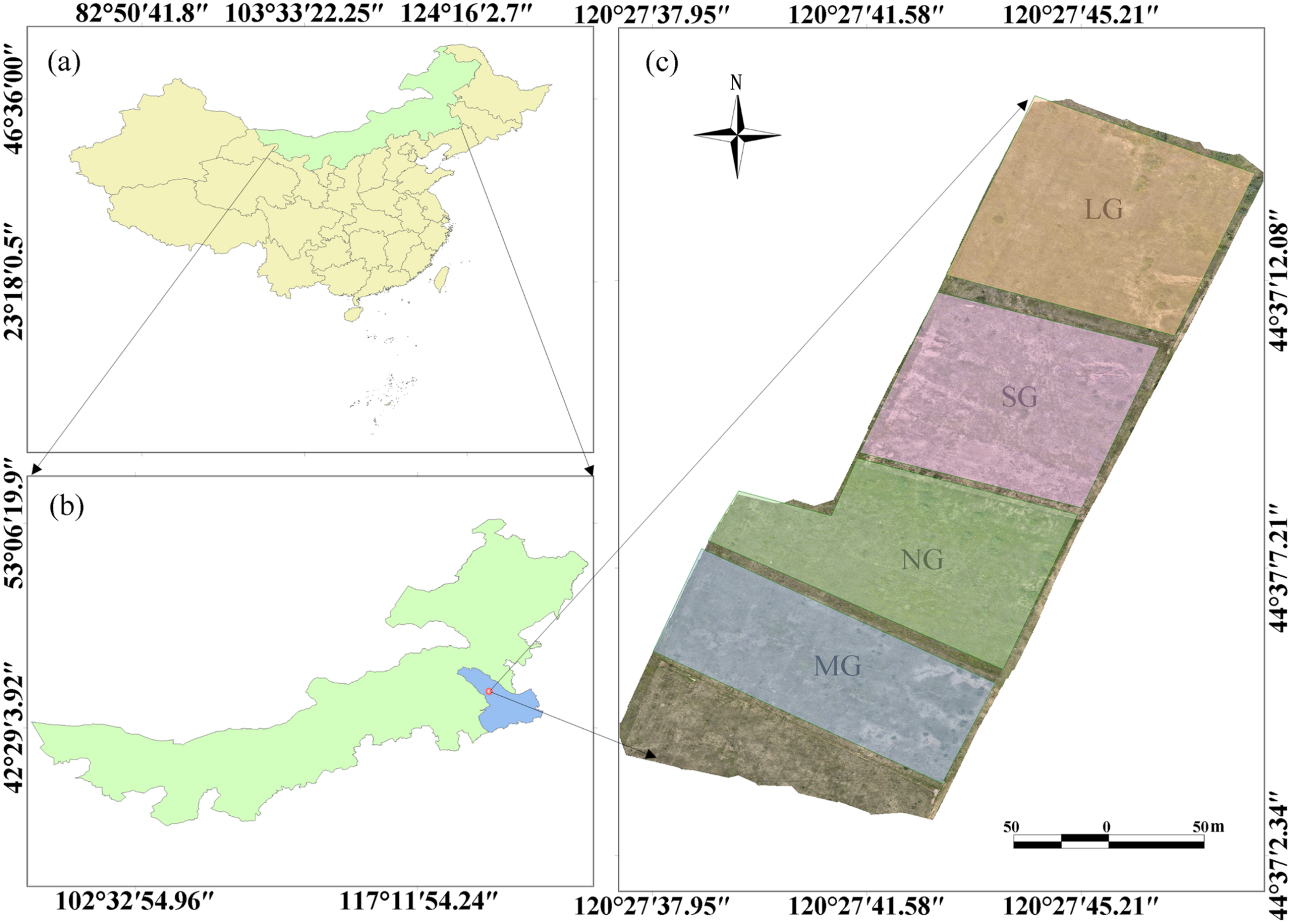
Location of the experimental sites and distribution of areas under different grazing intensities. **(a)** China, **(b)** Inner Mongolia, and **(c)** the study site. (e.g., NG, No Grazing; LG, Light Grazing; MG, Moderate Grazing; SG, Severe Grazing).

This study investigated the effects of varying grazing intensities on grassland ecosystems, commencing in June 2025. Grazing occurred from 15 June to 15 September, during which the sheep remained in their assigned subplots without interruption. The experimental site covered a total area of 3 ha, divided into four subplots of 0.75 ha each. A randomized block design was used to establish four grazing stocking rate treatments. Each subplot was grazed by 0, 3, 6, or 9 six-month-old rams, corresponding to 0, 4, 8, or 12 sheep units per ha, respectively. These treatments corresponded to no grazing (NG), light grazing (LG), moderate grazing (MG), and severe grazing (SG) (Zhang et al., 2024b). The overview of the experimental site and the distribution of the grazing intensity treatments are shown in **Figure 1**.

### 2.3 Data acquisition

#### 2.3.1 Ground investigation

Grassland technical personnel conducted ground surveys and established sample plots within experimental areas subjected to varying levels of grazing intensity. Sample plots were manually established for four grazing intensity levels—no grazing, light grazing, moderate grazing, and severe grazing—to identify herbaceous species influenced by grazing. Precise identification and extraction of regions of interest are allowed in the UAV imagery for subsequent analysis.

#### 2.3.2 Image collection and region of interest extraction

This study used a Mavic 3M UAV (SZ DJI Technology Co., Ltd., Shenzhen, China) to acquire imagery (Yu et al., 2023). The UAV is equipped with a visible light camera and a four-channel multispectral camera. The visible light camera features a 4/3-inch CMOS sensor with 20 million effective pixels. The multispectral camera captures four bands—green (560 ± 16 nm), red (650 ± 16 nm), red edge (730 ± 16 nm), and near-infrared (860 ± 26 nm)—with a resolution of 2592 × 1944 pixels per band. The UAV integrates a Global Navigation Satellite System (GNSS) with a Real-Time Kinematic (RTK) module, enabling centimeter-level positioning accuracy.

**Figure 2:**
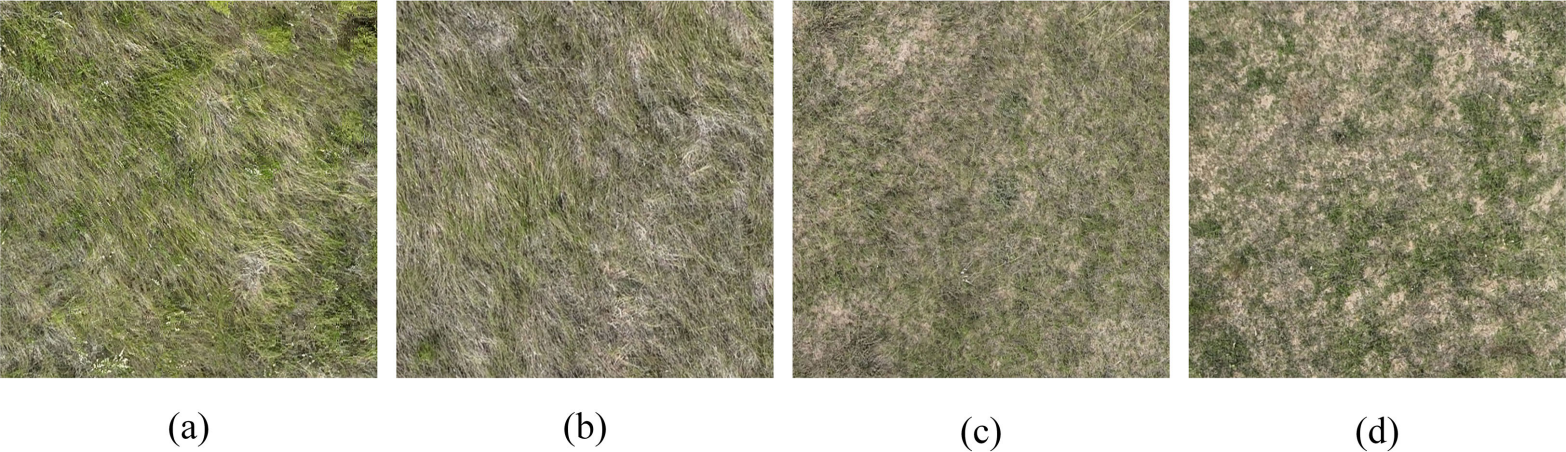
Visible-light UAV images illustrating different grazing intensities. **(a)** No grazing, **(b)** light grazing, **(c)** moderate grazing, and **(d)** severe grazing.

Imagery was acquired on 19 August 2025, between 11:00 and 12:00, at a flight altitude of 30 m. Both forward and side overlaps were set at 80% to ensure high-quality stitching and subsequent processing. A total of 1,536 visible and 3,155 multispectral images were acquired during the flight. Images were stitched using DJTerra software V3.0 (SZ DJI Technology Co., Ltd., Shenzhen, China), producing visible light orthoimages at ~0.87 cm/pixel and multispectral images at ~1.54 cm/pixel. RGB imagery corresponding to different grazing intensity areas is shown in **Figure 2**. After UAV image stitching, the red, green, and blue channels from the visible imagery, along with the green, red, red-edge, and near-infrared reflectance from the multispectral imagery, were extracted for the marked ground areas using ENVI 5.6 software (Harris Geospatial, Boulder, CO, USA)(Fenghua Yu et al., 2024). To ensure representativeness, region of interest (ROI) samples were selected using a stratified random sampling strategy within each grazing treatment subplot. A total of 720 samples were extracted, with 180 samples assigned to each of the four grazing intensity levels (NG, LG, MG, SG), ensuring balanced class distribution and consistent spatial coverage across treatments.

### 2.4 Grazing intensity monitoring method based on UAV multi-source remote sensing

**Figure 3:**
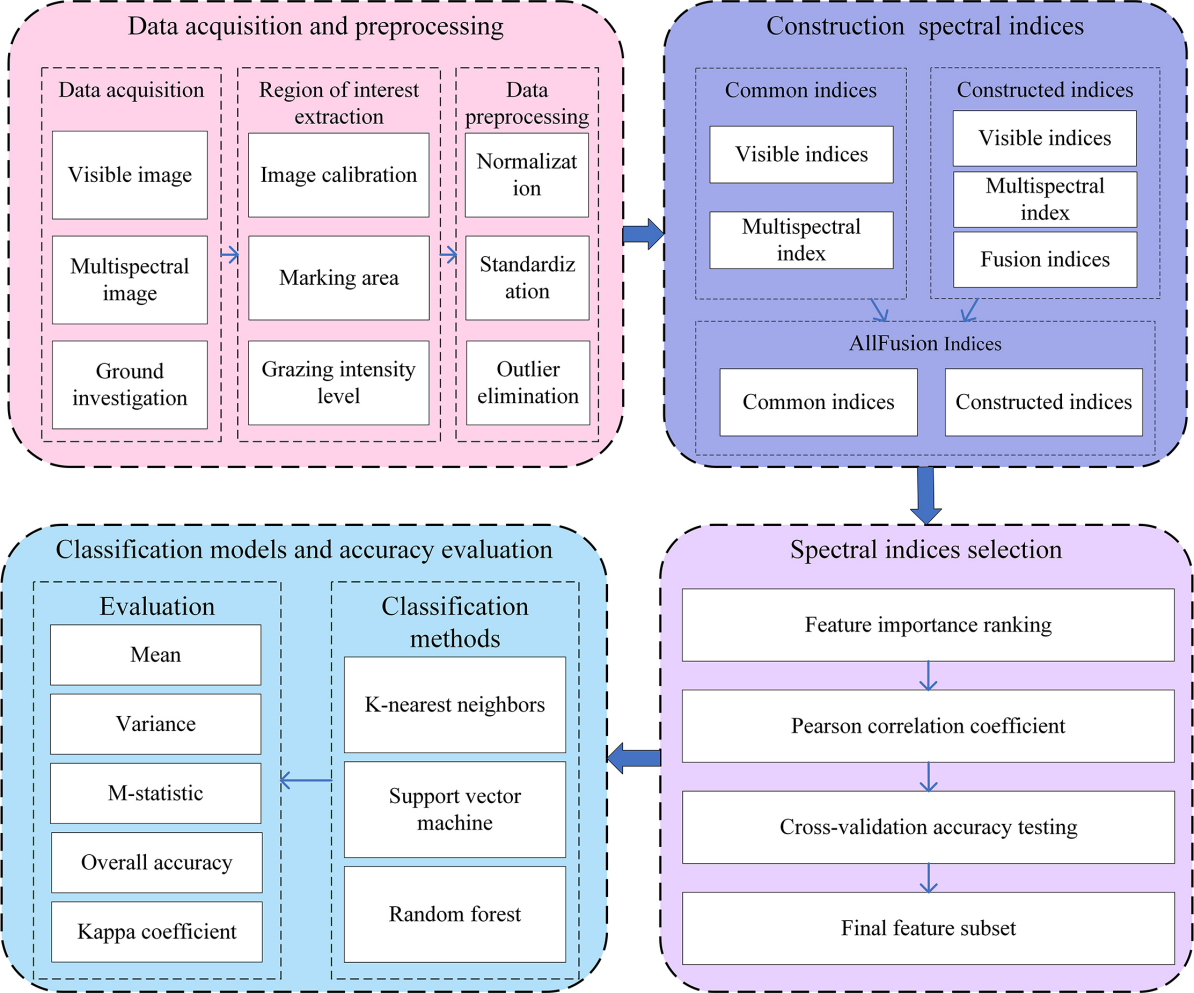
Schematic framework for monitoring grazing intensity using multi-source UAV imagery.

**Figure 3** illustrates the framework for monitoring grazing intensity using multi-source UAV imagery. The process mainly involves four steps: (1) data acquisition and preprocessing, (2) construction and fusion of multi-source SIs, (3) AIFS, and (4) classification modeling and accuracy evaluation. Data pre-processing includes normalization, standardization, and outlier removal. Multi-source SIs are derived from multiple features, integrating conventional and self-constructed indices to capture complementary information across data sources. An AIFS method, which combines feature importance assessment with redundancy removal, is employed to select the optimal combination of indices gradually. Finally, the performance of the optimized fused features is evaluated using various classification algorithms to achieve high accuracy and robust monitoring.

#### 2.4.1 Data preprocessing

To ensure balanced contributions from visible and multispectral data in subsequent multi-source feature fusion, the raw data were first preprocessed. The pixel values of the red ( R_RGB), green ( G_RGB), and blue ( B_RGB) bands in the visible imagery were normalized as follows (Cao et al., 2021):


$$R\_RGB_{norm}=\frac{R\_RGB}{R\_RGB+G\_RGB+B\_RGB}$$

$$G\_RGB_{norm}=\frac{G\_RGB}{R\_RGB+G\_RGB+B\_RGB}$$

$$B\_RGB_{norm}=\frac{B\_RGB}{R\_RGB+G\_RGB+B\_RGB}$$

The reflectance values of the green ( G_MS), red ( R_MS), red edge ( RE_MS), and near-infrared ( NIR_MS) bands in multispectral imagery did not require further normalization. This preprocessing lays the foundation for subsequent construction and fusion of multi-source SIs, preventing feature bias caused by differences in numerical scales.

#### 2.4.2 Multi-source SIs construction

SIs were calculated from combinations of different spectral bands to enhance target features, improve stability, and mitigate interference from factors such as illumination (Guan et al., 2022). Common approaches for constructing SIs include ratio-based, difference-based, and interaction-based combinations. In this study, 14 self-constructed indices were developed by integrating visible and multispectral band information, optimizing inter-band complementarity, and enhancing feature recognition. Both conventional and self-constructed SIs are summarized in **Table 1** (Varela et al., 2021) (Zhang et al., 2021b) (Tilly et al., 2015) (Bendig et al., 2015) (Liedtke et al., 2020) (Zhou et al., 2019) (Yue et al., 2019) (Pamungkas, 2023) (Zhou et al., 2019) (Pamungkas, 2023).

**Table 1:** Spectral indices used in this study.

Type	SI	Formula	Reference
Visible light SIs	ExG	2·G_RGB−R_RGB−B_RGB	(Varela et al., 2021)
	VARI	(G_RGB−R_RGB)/(G_RGB+R_RGB−B_RGB)	(Zhang et al., 2021b)
	ExGR	ExG−(1.4·R_RGB−G_RGB)·ExG − (1.4·R_RGB − G_RGB)	(Tilly et al., 2015)
	RGBVI	$\left(G\_RGB^{2}-\;R\_RGB\cdot B\_RGB)/(G\_RGB^{2}+\;R\_RGB\cdot B\_RGB\;\right)$	(Bendig et al., 2015)
Multispectral SIs	NDVI	(NIR_MS−R_MS)/(NIR_MS+R_MS)	(Liedtke et al., 2020)
	GNDVI	(NIR_MS−G_MS)/(NIR_MS+G_MS)	(Zhou et al., 2019)
	SAVI	(1+L)(NIR_MS−R_MS)/(NIR_MS+R_MS+L)	(Yue et al., 2019)
	MSAVI	$(2\cdot NIR\_MS+1-\sqrt{{\left(2\cdot NIR\_MS+1\right)}^{2}-8\times (NIR\_MS-R\_MS)})/2$	(Pamungkas, 2023)
	NDRE	(NIR_MS−RE_MS)/(NIR_MS+RE_MS)	(Zhou et al., 2019)
	EVI2	2.5·(NIR_MS−R_MS)/(NIR_MS+2.4·R_MS+1)	(Pamungkas, 2023)
Self-constructed SIs	idx1	G_RGB/(B_RGB)	This study
	idx2	G_RGB/(R_RGB)	This study
	idx3	NIR_MS/RE_MS	This study
	idx4	NIR_MS/R_MS	This study
	idx5	(NIR_MS−G_MS)/(NIR_MS+G_MS)	This study
	idx6	(E_MS−R_MS)/(RE_MS+R_MS)	This study
	idx7	$\left(B\_RGB_{norm}-RE\_MS)/(B\_RGB_{norm}+RE\_MS\right)$	This study
	idx8	$\left(\;G\_RGB_{norm}\cdot NIR\_MS)/(R\_MS\cdot RE\_MS\right)$	This study
	idx9	$\left(B\_RGB_{norm}\cdot NIR\_MS)/(R\_RGB_{norm}\cdot G\_RGB_{norm}\right)$	This study
	idx10	(R_MS·RE_MS)/(G_MS·NIR_MS)	This study
	idx11	R_RGB/(R_RGB+G_RGB+B_RGB)	This study
	idx12	G_RGB/(R_RGB+G_RGB+B_RGB)	This study
	idx13	B_RGB/(R_RGB+G_RGB+B_RGB)	This study
	idx14	$\left(NIR\_MS-mean(RGB_{norm}))/(NIR\_MS+mean(RGB_{norm})\right)$	This study

#### 2.4.3 Automatic incremental feature selection

To further enhance the discriminative power of the integrated features, this study employed an AIFS method on the feature matrix ${\text{X}}_{\text{all}}$, which combines visible indices, multispectral indices, and self-constructed indices.

##### 2.4.3.1 Feature importance ranking

Feature importance was evaluated using the Random Forest (RF) algorithm (Belgiu and Drăguţ, 2016). Let the feature fusion matrix be denoted as ${\text{X}}_{all}\in {\mathbb{R}}^{n\times m}$, where n is the number of samples and m is the total number of features, and let the class vector be ${\text{Y}}_{all}$. The RF model builds multiple decision trees and calculates the incremental prediction error on out-of-bag (OOB) samples for each tree to obtain the importance score $I_{j}$ for each feature:


$$I_{j}=\frac{1}{T}\overset{T}{\underset{t=1}{\sum }}\Delta {\text{Err}}_{t,j}^{OOB}$$

Here, *T* is the total number of decision trees, and $\Delta {\text{Err}}_{t,j}^{OOB}$ represents the increase in OOB error caused by randomly permuting the values of feature *j* in the *t*-th tree. Features were then ranked in descending order based on $I_{j}$, resulting in the sorted feature matrix $X_{sorted}$.

##### 2.4.3.2 Incremental feature selection for combinations

To minimize the impact of redundant features while maintaining classification discriminative power, an incremental combination strategy was used to select features gradually. The procedure is as follows:

1) Candidate features were sequentially added from the sorted feature matrix.

2) The Pearson correlation coefficient between the candidate feature $x_{j}$ and the selected feature set *S* was calculated (Guan et al., 2023):


$$\rho_{j,k}=\frac{\text{cov}(x_{j},x_{k})}{\sigma_{x_{j}}\sigma_{x_{k}}},\;\;k\in S$$

Suppose the absolute Pearson correlation $|\rho_{j,k}|$ between the candidate feature $x_{j}$ and any feature $x_{k}$ in the selected set *S* exceeds the threshold $\rho_{thresh}$=0.8. In that case, the candidate feature is considered highly correlated with existing features and is excluded. Otherwise, it is added to the selected feature set.

3) For each newly added feature, overall accuracy (OA) was calculated using 5-fold cross-validation:


$$\text{OA}=1-\text{CV Loss}=1-\frac{1}{K}\overset{K}{\underset{i=1}{\sum }}L_{i}$$

Here, $L_{i}$ denotes the error rate for the *i*-th fold, with K=5. Newly added features were retained in the final subset only if they improved the OA. This strategy ensured that the selected features minimized redundancy while maximizing classification performance.

##### 2.4.3.3 Output the final feature subset

After iterative incremental selection, the optimal feature subset, $X_{constructed}$, is obtained. This subset serves as input for subsequent grazing intensity classification, enabling high-precision and robust classification performance.

**Algorithm 1** summarizes the AIFS procedure. The algorithm ranks features using RF importance, incrementally evaluates candidate features based on Pearson correlation and cross-validated accuracy, and retains only those that improve classification while minimizing redundancy.

Algorithm 1

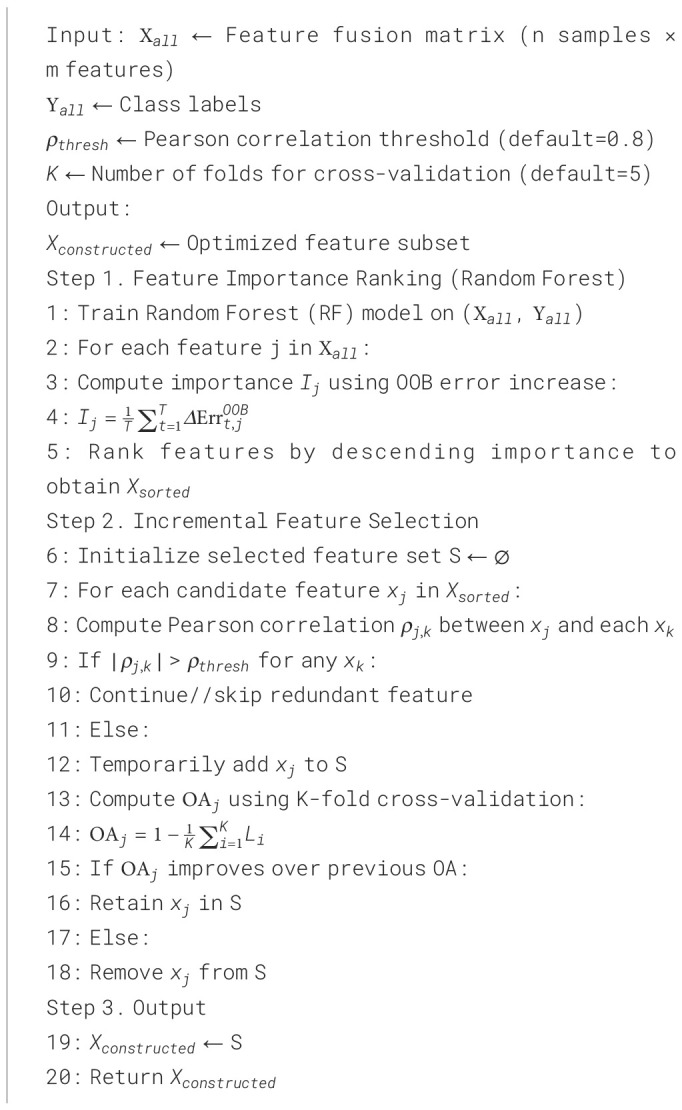

#### 2.4.4 Classification methods

This study used three widely applied classification algorithms—K-nearest neighbor (KNN) (Abdullah et al., 2001), support vector machine (SVM) (Guan et al., 2025), and Random Forest (RF) (Guo et al., 2024)—to classify grazing intensity based on the fused features.

##### 2.4.4.1 K-nearest neighbor

KNN is a non-parametric classification method based on the similarity of samples. For a sample $x_{0}$, the algorithm calculates distances to all training samples, selects the K nearest neighbors, and assigns $x_{0}$ to the majority class of these neighbors. The procedure is expressed as:


$$\hat{y}=\underset{c}{\arg\max}\underset{i\in N_{K}(x_{0})}{\sum }\omega_{i}\cdot I(y_{i}=c)$$

Here, $N_{K}(x_{0})$ denotes the set of K nearest neighbors of $x_{0}$, $\omega_{i}=1/\text{d}(x_{0},x_{i})$ represents the distance weight, and I(·) is the indicator function.

##### 2.4.4.2 Support vector machine

SVM is a maximum-margin method for binary or multi-class classification, separating samples by finding the optimal hyperplane that maximizes the margin between classes. In this study, a radial basis function (RBF) kernel was used for non-linear mapping. Kernel scale and box constraint parameters were optimized via grid search. The SVM classifier is expressed as:


$$f(x)=\text{sign}(\overset{N}{\underset{i=1}{\sum }}\alpha_{i}y_{i}K(x_{i},x)+b)$$

Here, $K(x_{i},x)$ denotes the RBF kernel; $\alpha_{i}$ is the Lagrange multiplier; and *b* is the bias term.

##### 2.4.4.3 Random forest

Random Forest (RF) is an ensemble learning method that classifies data by constructing multiple decision trees. Each tree is trained using bootstrap sampling, which randomly selects a subset of features at each node, thereby reducing overfitting and improving stability. Final classification is determined by majority voting:


$$\hat{y_{0}}=\text{mode}\{h_{1}(x0),h_{2}(x0),\ldots ,h_{T}(x0)\}$$

Here, $h_{t}$ is the prediction of the *t*-th decision tree, and T is the total number of trees.

##### 2.4.4.4 Classification test and parameter setting

To ensure balanced training and test sets, the dataset was divided using the Sample set Partitioning based on joint X–Y distances (SPXY) method, with 70% allocated to training and 30% to testing (Wu et al., 1996). Grid search optimization was performed for all classifiers. For KNN, the number of neighbors K was set to [3, 5, 7, 9] with distance metrics including Euclidean, Manhattan, and cosine. For SVM, the RBF kernel scale was set to [0.1, 0.5, 1, 2], and the box constraint parameter C was set to [0.1, 1, 10]. For RF, the number of trees was set to [100, 200, 300], with minimum leaf nodes [1, 5, 10].

#### 2.4.5 Evaluation indicators

##### 2.4.5.1 Separability evaluation

To assess the discriminative capability of spectral features under different grazing intensities, a qualitative analysis was first performed by comparing means and variances. Quantitative assessment was conducted using one-way analysis of variance (ANOVA) followed by Tukey’s multiple comparison test (Stoline, 1981). To quantify the classification ability of SIs, the M statistic was introduced, defined as: $M=\frac{\left|\bar{x_{1}}-\bar{x_{2}}\right|}{s_{1}^{2}+s_{2}^{2}}$Here, $\bar{x_{1}}$ and $\bar{x_{2}}$ denote the sample means of the two categories, and $s_{1}^{2}$ and $s_{2}^{2}$ represent the corresponding within-class variances. A higher M value indicates a greater difference in feature distribution between the two categories, reflecting stronger separability (Smith et al., 2007).

##### 2.4.5.2 Evaluation of classification accuracy

After fitting the classifier to the training set, its performance was evaluated on the test set using overall accuracy (OA) and the Kappa coefficient ( κ) (Guan et al., 2022). Overall OA was calculated as:


$$\text{OA}=\frac{\sum_{i=1}^{k}c_{ii}}{\text{n}}$$

Here, *k* represents the total number of categories, and $\sum_{i=1}^{k}c_{ii}$ represents the number of correctly classified samples.

The Kappa coefficient ( κ) measures the agreement between the observed classification accuracy and that expected by random chance, calculated as:


$$\kappa =\frac{p_{o}-p_{e}}{1-p_{e}}$$

Here,


$$p_{o}=OA\;,\;p_{e}=\frac{\sum_{i=1}^{k}(\sum_{j=1}^{k}c_{ij})(\sum_{j=1}^{k}c_{ji})}{n^{2}}$$

Here, $p_{o}$ denotes the observed classification accuracy, and $p_{e}$ represents the expected accuracy under random classification.

## 3 Results

### 3.1 Detection of grazing intensity by spectral features

**Figure 4:**
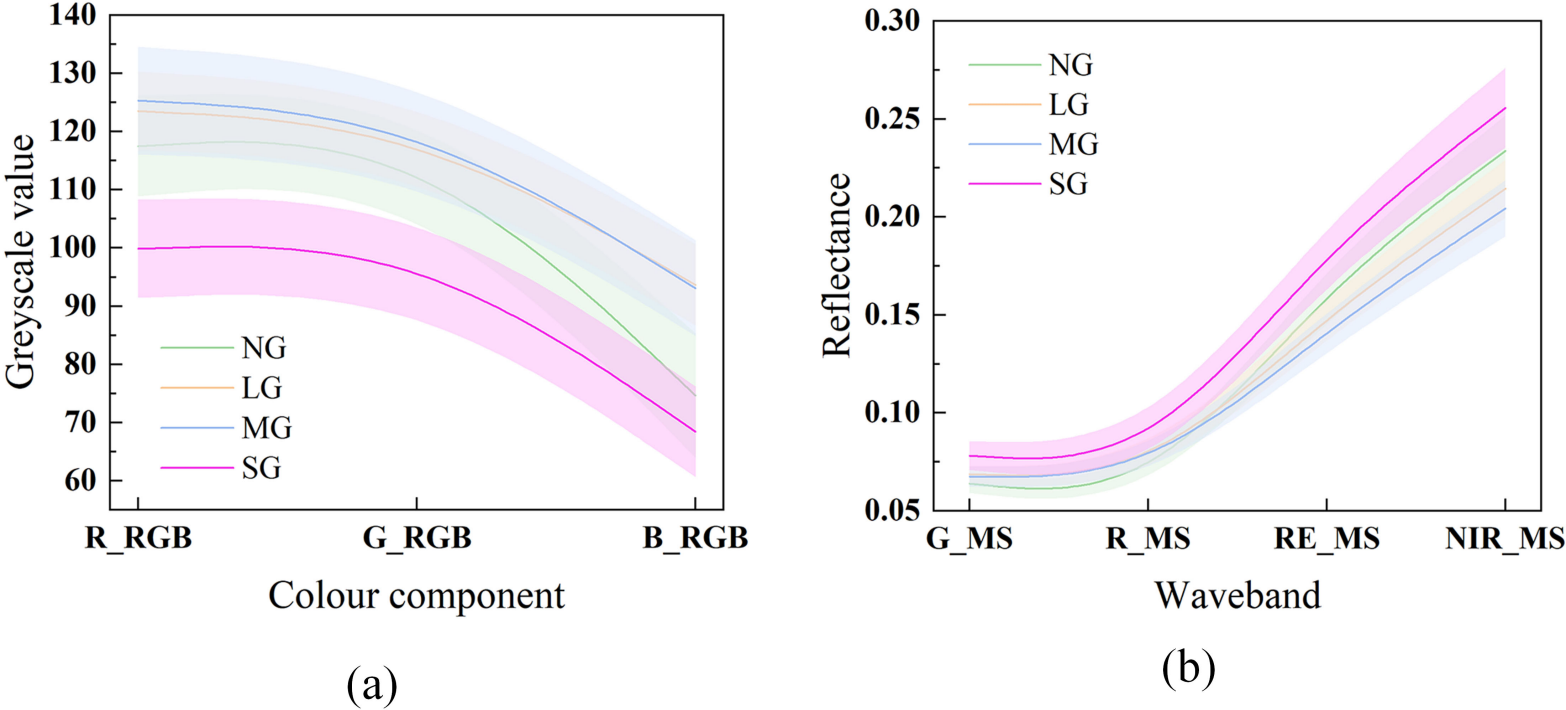
Mean and variance curves under different grazing intensities. **(a)** Visible-light features, and **(b)** multispectral features. (e.g., NG, No Grazing; LG, Light Grazing; MG, Moderate Grazing; SG, Severe Grazing).

The spectral mean and variance curves under different grazing intensities are presented in **Figure 4**. The grey values of the three visible-light features generally increased with grazing intensity, but decreased under severe grazing. In the multispectral bands, green and red reflectance increased with grazing intensity, whereas the red-edge and near-infrared bands showed decreasing trends. However, under severe grazing, both the red-edge and near-infrared bands exhibited an increasing trend.

**Figure 5:**
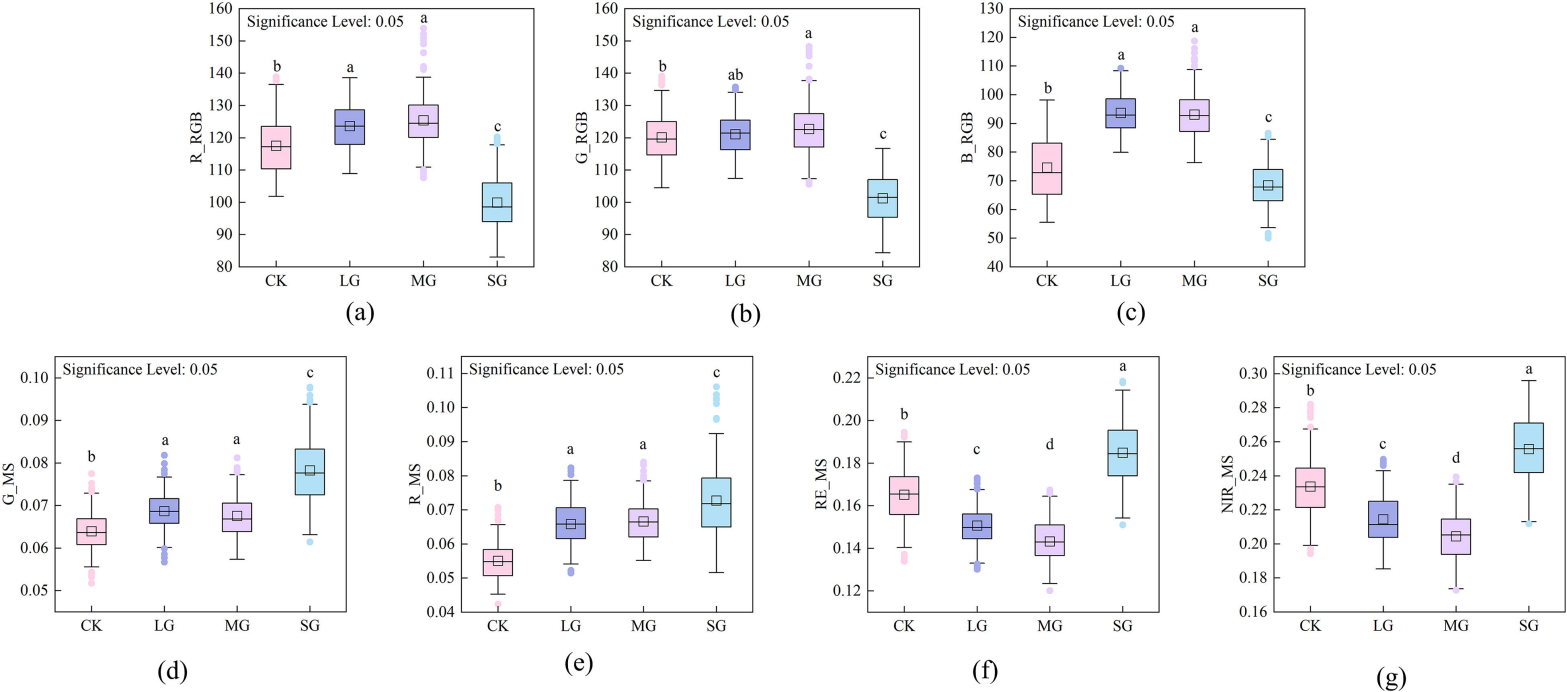
Tukey’s multiple comparison results for original spectral features across grazing intensities. **(a)** R_RGB, **(b)** G_RGB, **(c)** B_RGB, **(d)** G_MS, **(e)** R_MS, **(f)** RE_MS, and **(g)** NIR_MS. Significant differences among grazing groups are indicated (p < 0.05). (e.g., NG, No Grazing; LG, Light Grazing; MG, Moderate Grazing; SG, Severe Grazing).

ANOVA results for visible and multispectral features revealed significant differences (p < 0.05) across grazing intensities, indicating that grazing intensity has a substantial impact on spectral responses. Tukey’s *post-hoc* test (**Figure 5**) revealed that the red ( R_RGB) and blue ( B_RGB) components significantly distinguished between low, medium, and severe grazing intensities, with the most substantial differences observed under extreme grazing conditions. The green ( G_RGB) component demonstrated a limited ability to separate low and medium grazing intensities, but retained significant differentiation under severe grazing conditions. For multispectral bands, the green ( G_MS) and red ( R_MS) bands showed substantial differences between low and severe grazing intensities. The red-edge ( RE_MS) and near-infrared ( NIR_MS) bands were sensitive not only to the transition between low and severe grazing, but also to the transition between medium and severe grazing, highlighting their strong ability to capture variations in vegetation status and grazing conditions. Overall, multispectral features offered more comprehensive discrimination of grazing levels than visible-light features. Specifically, the red-edge and near-infrared bands effectively captured vegetation changes under medium-to-severe grazing, whereas visible-light features were more sensitive to extreme grazing.

**Figure 6:**
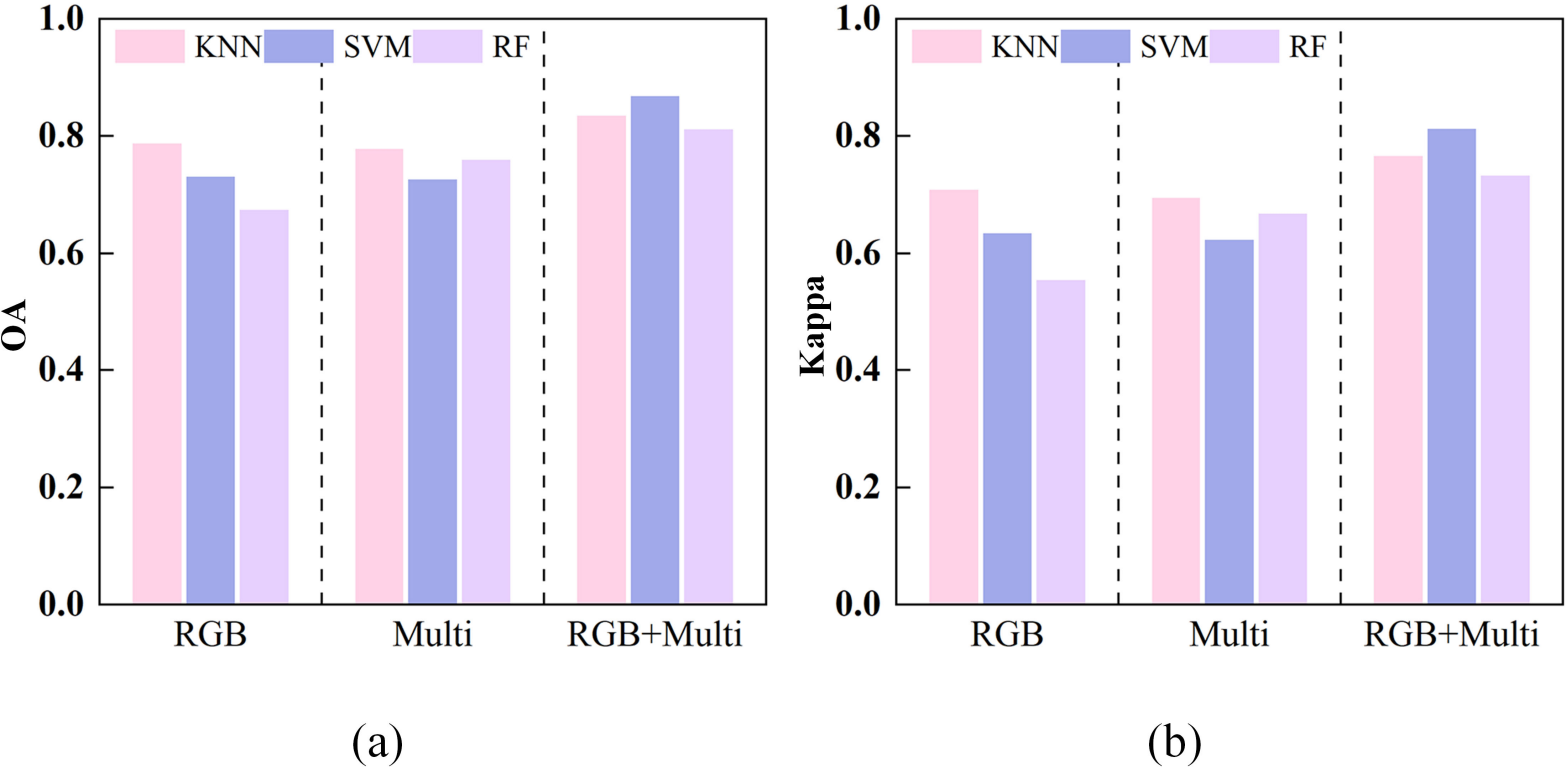
Classification performance of three feature sets (RGB, Multi, and RGB+Multi) using KNN, SVM, and RF classifiers. **(a)** OA, and **(b)** Kappa coefficient.

In this study, the classification performance of grazing intensity was evaluated using KNN, SVM, and RF based on three feature sets: visible light (RGB), multispectral (Multi), and multi-source fusion (RGB+Multi). The results are presented in **Figure 6**. Among single-source features, RGB achieved the highest accuracy with KNN, yielding an OA of 78.77% and a Kappa of 70.86%. The Multi-features performed best with RF, achieving an OA of 75.94% and a Kappa of 66.74%. In contrast, the multi-source fusion features markedly improved classification, achieving OA values of 83.49%, 86.79%, and 81.13%, with corresponding Kappa values of 76.62%, 81.28%, and 73.25%. These results demonstrate that integrating visible and multispectral features leverages complementary spectral information to improve discrimination among grazing intensity levels. Overall, the combination of multi-source fusion features with the SVM classifier yielded the highest accuracy and the most consistent results.

**Figure 7:**
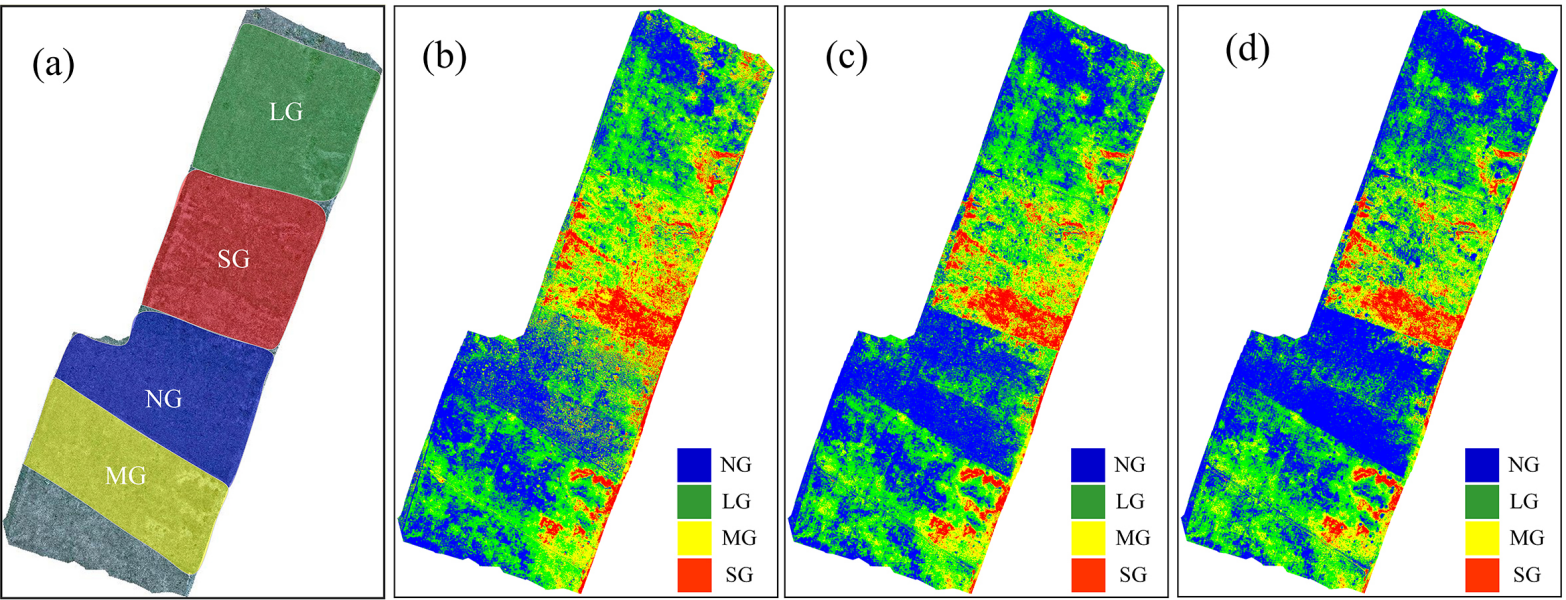
Pixel-level classification maps under different combinations of original features and classifiers. **(a)** Reference grazing intensity map; **(b)** RGB features with KNN, **(c)** multispectral (Multi) features with KNN, and **(d)** multi-source fusion (RGB+Multi) features with SVM. (e.g., NG, No Grazing; LG, Light Grazing; MG, Moderate Grazing; SG, Severe Grazing).

**Figure 7** presents pixel-level classification results under different combinations of original features and classifiers. As shown in **Figure 7a**, the experimental area was divided into four grazing intensity levels: no grazing (NG), light grazing (LG), moderate grazing (MG), and severe grazing (SG). **Figures 7b–d** illustrate the classification maps generated by different feature combinations and classifiers. The classification map based on visible (RGB) features using the KNN classifier (**Figure 7b**) roughly captures the grazing intensity distribution but shows some confusion between LG and MG regions. When using multispectral (Multi) features with the KNN classifier (**Figure 7c**), the classification accuracy improves slightly, particularly in distinguishing NG and SG areas. However, misclassifications remain at moderate levels. In contrast, the multi-source fusion (RGB+Multi) combined with the SVM classifier (**Figure 7d**) produces the most accurate and spatially consistent classification, with clear boundaries between different grazing intensities that closely align with the actual distribution. This demonstrates that multi-source feature fusion effectively enhances spatial discrimination of grazing intensity and mitigates spectral confusion caused by soil–vegetation mixing in sandy grasslands.

### 3.2 Detection of grazing intensity by spectral indices

**Figure 8:**
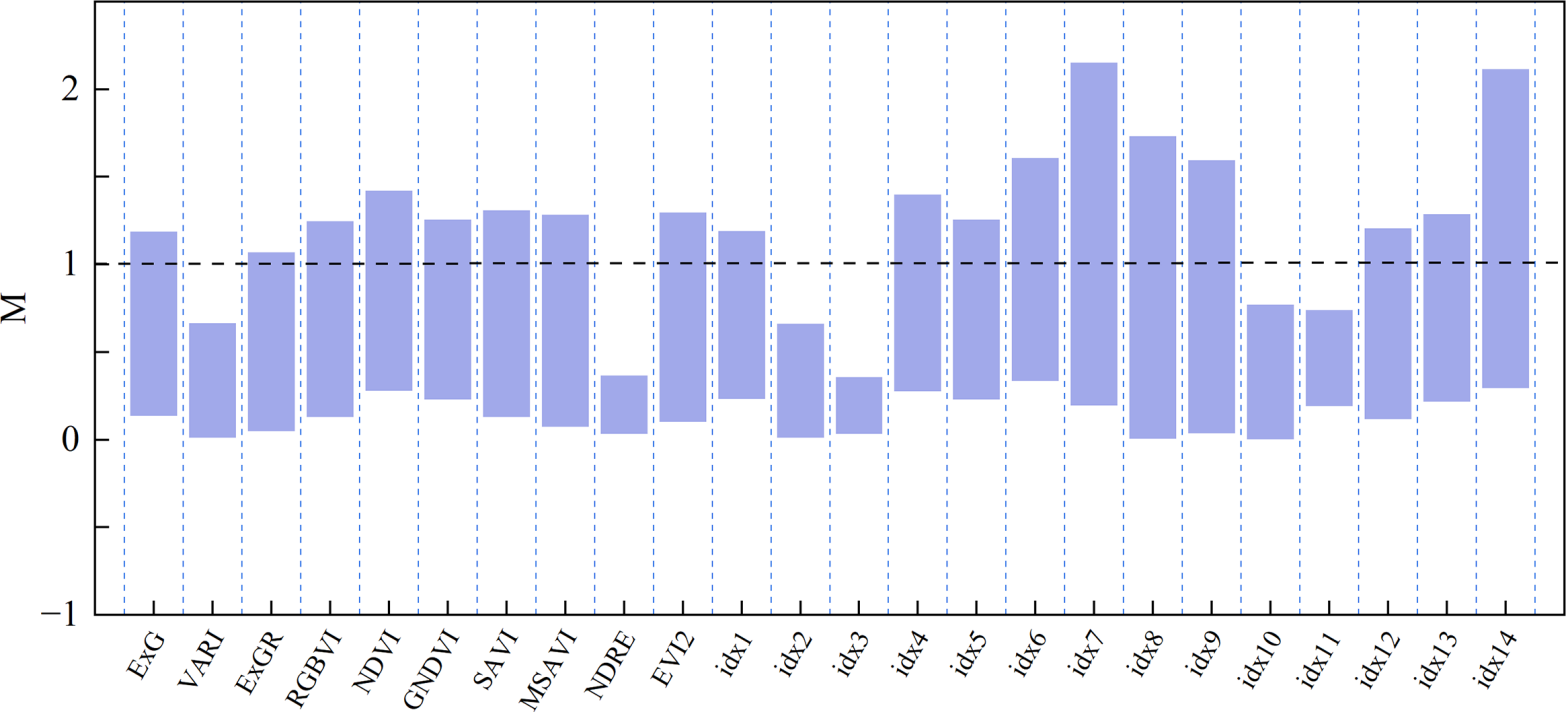
Separability of spectral indices evaluated using the M statistic.

The M-statistic was applied to assess the separability of the SIs, with results presented in **Figure 8**. For most indices, M values exceeded 1, indicating good separability among grazing intensity levels. Traditional visible indices (ExG, ExGR, RGBVI) and red–NIR indices (NDVI, GNDVI, SAVI, MSAVI, EVI2) generally exhibited high separability. Their maximum M values all exceeded 1, while the mean values were mainly within the range of 0.6 to 1.0. In contrast, NDRE, idx3, and several other indices showed poor separability, with maximum M values below 0.4. Notably, among the self-constructed indices, idx6, idx7, and idx14 performed best, with maximum M values exceeding 1.5. In particular, idx7 and idx14 reached extreme values above 2, demonstrating substantial advantages in capturing vegetation responses to grazing intensity and outperforming traditional indices.

**Figure 9:**
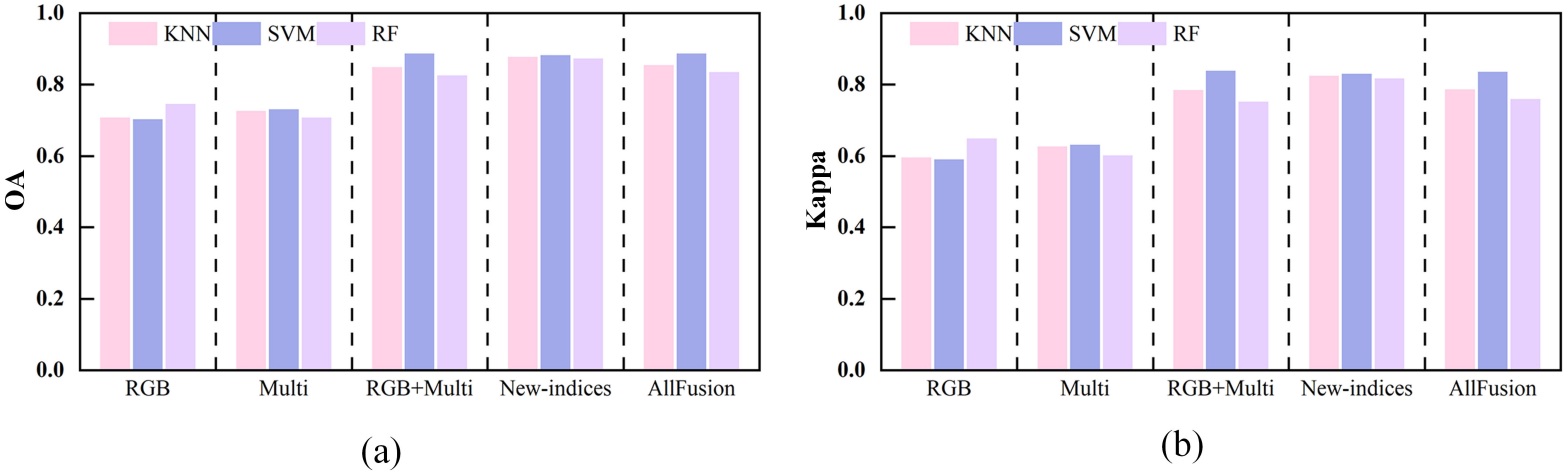
Classification performance for different combinations of spectral indices across three classifiers. **(a)** OA, and **(b)** Kappa coefficient.

**Figure 9** presents the classification results of three classifiers (KNN, SVM, and RF) using different combinations of SIs. The classification accuracy of visible indices (RGB) was comparable to that of multispectral indices (Multi), with Multi showing slightly better overall performance. The results indicate that near-infrared and red-edge bands provide stronger discriminative capability for grazing level identification. Integrating RGB and Multi (RGB+Multi) markedly improved accuracy, with OA values of 84.91%, 88.68%, and 82.55% for KNN, SVM, and RF, respectively. Corresponding Kappa coefficients all exceeded 75%, indicating strong complementarity between visible and multispectral features. With the newly constructed indices (New-indices), classification performance further improved: all three classifiers achieved OA values above 87% and Kappa values above 82%. These results demonstrate that the newly proposed indices more effectively capture differences in grazing intensity and enhance class separability. Under the AllFusion condition (RGB+Multi+New-indices), results were comparable to those from RGB+Multi and New-indices. Overall, SVM showed the most consistent performance, with a maximum OA of 88.68% and a Kappa above 83%. Comparative analysis indicates that the newly constructed indices substantially improve discrimination of grazing intensity, outperforming single-band combinations, while multi-source feature fusion further enhances model robustness.

**Figure 10:**
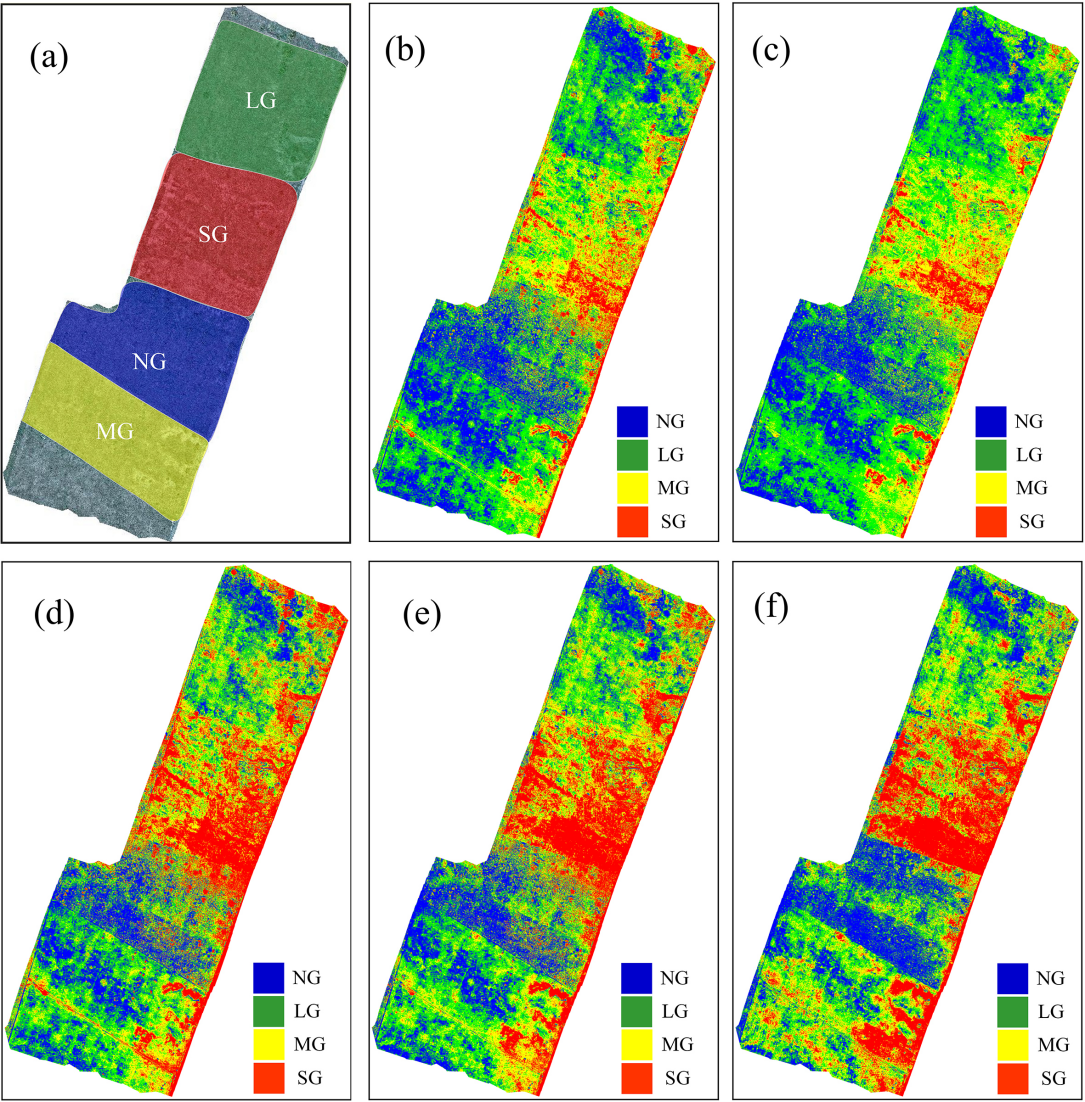
Pixel-level classification maps derived from different spectral-index combinations and classifiers. **(a)** Reference grazing intensity map, **(b)** RGB-derived indices with RF, **(c)** Multi-derived indices with SVM, **(d)** RGB+Multi indices with SVM, **(e)** New indices set with SVM, and **(f)** AllFusion with SVM. (e.g., NG, No Grazing; LG, Light Grazing; MG, Moderate Grazing; SG, Severe Grazing).

**Figure 10** presents pixel-level classification maps generated from different SI sets and classifiers. **Figure 10a** shows the reference grazing-intensity partition (NG, LG, MG, SG). **Figure 10b-f** display classification maps produced using different index sets and the best-performing classifier for each set. The map produced from RGB-derived indices with RF (**Figure 10b**) captures the broad spatial pattern but shows misclassification between adjacent LG and MG patches. Using multispectral indices with SVM (**Figure 10c**) improves discrimination of NG and SG zones and reduces local speckle. The fused RGB+Multi set under SVM (**Figure 10d**) increases spatial continuity and sharpens boundaries. Notably, the AllFusion map (**Figure 10f**), which uses the full index suite, attains the best overall spatial agreement with the reference—reflecting the benefit of combining complementary information across index families. The map based on the newly constructed indices (**Figure 10e**) also yields high spatial consistency and particularly good detection of moderate grazing, despite using a more compact feature set. Overall, AllFusion and the new-index set both perform strongly: AllFusion slightly surpasses other sets in overall spatial agreement, while the new indices offer nearly comparable accuracy with fewer features, demonstrating a favorable trade-off between completeness and parsimony.

### 3.3 Detection of grazing intensity by feature selection

This study applied multiple feature selection methods for full-feature fusion, including random frog (RF) (Li et al., 2012), ReliefF (Robnik-Šikonja and Kononenko, 2003), least-squares mutual information (LSMI) (Suzuki et al., 2009), successive projections algorithm (SPA) (Araújo et al., 2001; Yu et al., 2020), elimination of uninformative variables (UVE) (Centner et al., 1996), competitive adaptive reweighted sampling (CARS) (Li et al., 2009), and AIFS. Each method used its own scoring or importance distribution to determine the selected features automatically. As shown in **Table 2**, the number and type of features selected by different methods varied slightly but showed overall consistency. Most methods consistently selected traditional vegetation indices (e.g., ExG, GNDVI, NDRE, RGBVI, MSAVI) and several self-constructed indices (e.g., idx7, idx8, idx9, idx14), suggesting that these features have strong discriminatory power for grazing intensity across different algorithms. Among these, idx7, idx9, and idx14 appeared most frequently across the six methods, highlighting their stability and central importance. In contrast, the RF and AIFS methods selected fewer features.

**Table 2:** Spectral indices selected by different feature selection methods.

Method	Number of indices	Specific spectral indices
RF	7	idx1, idx6, idx7, idx8, idx9, idx13, idx14
ReliefF	10	ExG, GNDVI, NDRE, idx3, idx5, idx6, idx7, idx8, idx9, idx14
LSMI	12	ExG, RGBVI, MSAVI, idx1, idx3, idx5, idx6, idx7, idx8, idx9, idx11, idx14
SPA	12	VARI, ExGR, GNDVI, MSAVI, NDRE, idx1, idx4, idx7, idx8, idx9, idx10, idx11
UVE	11	ExG, ExGR, RGBVI, idx1, idx2, idx3, idx4, idx7, idx9, idx10, idx14
CARS	11	VARI, RGBVI, GNDVI, NDRE, idx3, idx4, idx6, idx7, idx9, idx10, idx14
AIFS	7	idx9, idx8, idx7, NDVI, idx2, idx3, NDRE

**Table 3** shows that various feature selection methods exhibit distinct performances across different classifiers (KNN, SVM, RF). Overall, the AIFS method produced the best classification results. SVM achieved high accuracy, with OA and Kappa values of 92.13% and 88.99%, respectively, and also showed strong performance under KNN and RF. This result demonstrates that the method achieves optimal discrimination while requiring fewer features. In contrast, the AllFusion approach, which utilizes all features, yielded the lowest accuracy. The OA and Kappa values across all three classifiers were significantly lower than those of other methods, indicating that redundant features not only fail to improve performance but may also impair classification effectiveness. Overall, appropriate feature selection substantially improves the accuracy of grazing intensity classification, with the AIFS method providing both efficiency and precision.

**Table 3:** Classification results of feature selection methods under different classifiers.

Method	KNN		SVM		RF	
	OA (%)	Kappa (%)	OA (%	Kappa (%)	OA (%)	Kappa (%)
RF	87.96	83.14	87.04	81.90	85.65	79.87
ReliefF	89.35	84.95	90.28	86.23	87.50	82.27
LSMI	89.82	85.23	88.43	83.15	85.65	79.08
SPA	86.11	79.81	87.96	82.47	86.57	80.57
UVE	87.50	81.82	87.50	81.80	84.72	77.72
CARS	86.11	80.29	88.89	84.15	84.72	78.18
AIFS	91,20	87.72	**92.13**	**88.99**	87.96	83.17
AllFusion	83.49	76.63	86.79	81.28	81.13	73.25

Bold values indicate the highest accuracy and Kappa coefficient among all combinations.

**Figure 11:**
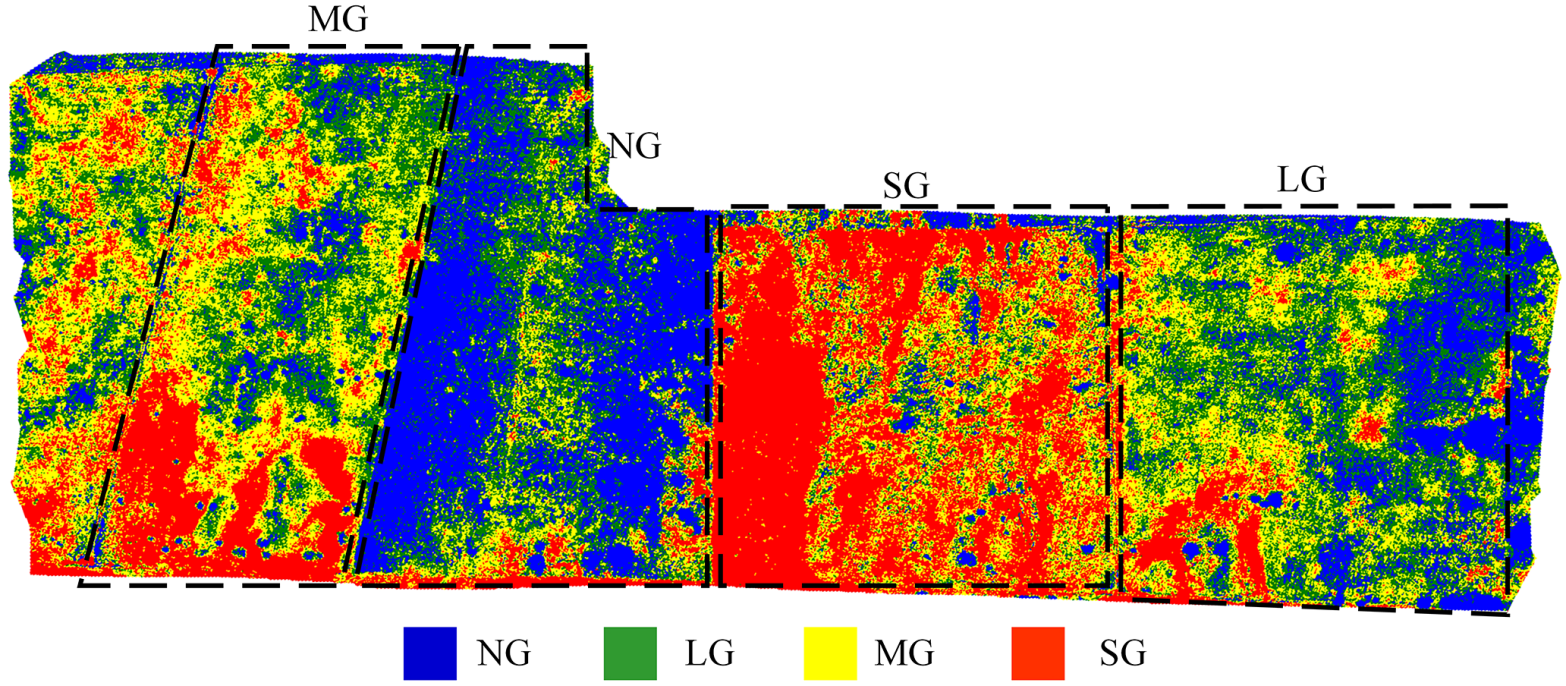
Pixel-level classification map of grazing intensity. (e.g., NG, No Grazing; LG, Light Grazing; MG, Moderate Grazing; SG, Severe Grazing).

The classification model developed in this study was applied to perform pixel-level classification on images generated by fusing visible and multispectral data. To reduce noise, a 3 × 3 filter was used to smooth the classification map (Guan et al., 2025), as shown in **Figure 11**. The predicted grazing intensities (blue, green, yellow, and red areas) show a clear correspondence with the ground-truth grazing intensity areas (dashed regions representing NG, LG, MG, and SG). Regions with actual grazing intensities of NG, LG, MG, and SG were predominantly predicted as blue, green, yellow, and red, respectively. These results indicate that the model can accurately predict varying grazing intensities, demonstrating robust overall performance. However, minor deviations exist between some local predictions and actual conditions, likely due to factors such as terrain undulations and the grazing behavior of sheep.

## 4 Discussion

This study investigated the remote sensing-based monitoring of grazing intensity in sandy grasslands by integrating visible and multispectral data acquired from UAVs with spectral indices and an automatic incremental feature selection (AIFS) method. The results demonstrate that multi-source data fusion and feature optimization substantially improve the discrimination of grazing intensities, with the SVM classifier achieving the highest accuracy (OA=92.13%, Kappa=88.99%). These findings highlight that, in complex ecosystems such as sandy grasslands—characterized by sparse vegetation and strong soil background effects—the integration of UAV multi-source imagery with intelligent feature selection effectively addresses the limitations of traditional approaches and provides a robust methodological framework for grassland ecological monitoring.

### 4.1 Spectral response characteristics and ecological interpretation

The spectral mean curves revealed that visible bands generally increased with grazing intensity but declined under severe grazing conditions. This nonlinear pattern can be explained by the pronounced soil–vegetation hybrid effect typical of sandy grasslands. Light to moderate grazing increases soil exposure, enhancing reflectance in blue, green, and red bands due to the bright sandy background. Under heavy grazing, however, trampling induces surface crusting and litter accumulation, which reduce overall reflectance. In the multispectral region, the green and red bands similarly increased with grazing intensity, while the red-edge and near-infrared (NIR) bands decreased, reflecting reductions in chlorophyll content and canopy integrity. The slight rebound of red-edge and NIR reflectance under severe grazing likely arises from soil high-albedo effects and the persistence of grazing-tolerant species.

These results differ from the monotonic spectral decline patterns reported in humid or dense grasslands (Oliveira et al., 2019; Zhang et al., 2021a), illustrating that vegetation spectral responses in semi-arid grasslands are co-regulated by physiological degradation of plants and enhanced background reflectance from soil. This interactive mechanism underscores the need to account for both vegetation and soil components when interpreting spectral responses in dryland ecosystems (Shi et al., 2021).

### 4.2 Performance of spectral indices and feature selection

Statistical analyses (ANOVA and Tukey’s tests) confirmed that all spectral indices exhibited significant differences among grazing intensities. Traditional vegetation indices (NDVI, GNDVI) effectively captured the decline in vegetation cover and photosynthetic activity between light and heavy grazing but were less sensitive to moderate grazing levels. Indices that integrate red and NIR information (NDRE, MSAVI) showed superior discrimination between low–moderate and moderate–heavy grazing intensities, aligning with previous findings from semi-arid regions (Hernandez et al., 2024; Pádua et al., 2024; Pranga et al., 2024).

The self-developed indices (idx7, idx9, idx14) demonstrated even greater adaptability and stability, reflecting the advantages of region-specific spectral design tailored to sandy grasslands. Feature selection consistently identified ExG, NDRE, MSAVI, and the newly constructed indices (idx7, idx9, idx14) as key predictors, confirming their robustness for grazing intensity monitoring (Xu et al., 2022).

Interestingly, NDRE and idx3 exhibited relatively low M-statistic values (<0.4), suggesting weak separability when evaluated independently. However, both were included in the final feature subset derived through AIFS. This apparent discrepancy highlights the difference between univariate separability and multivariate synergy. While the M-statistic evaluates features in isolation, the AIFS approach optimizes combinations based on their joint contribution to classification accuracy. Thus, even indices with limited standalone performance can provide complementary information that enhances the discriminative power and stability of the final model. The inclusion of correlation constraints further ensures non-redundancy among selected features, reinforcing the unique role of each feature in the optimized subset.

In addition, compared with commonly used feature selection algorithms such as ReliefF and CARS, AIFS also shows practical advantages in computational efficiency. ReliefF requires repeated distance-based sampling across the entire feature space, which becomes increasingly expensive as feature dimensionality grows. CARS relies on iterative Monte-Carlo sampling and partial-least-squares regression, making its computational burden dependent on repeated model fitting. In contrast, AIFS performs a single-pass importance ranking using Random Forests followed by lightweight incremental evaluation with correlation filtering and cross-validation. Because redundant features are removed early during correlation screening, subsequent computations are substantially reduced. As a result, AIFS generally achieves competitive or higher classification accuracy while requiring fewer iterations and fewer full model evaluations than ReliefF and CARS, making it more suitable for UAV-based multi-source datasets where dozens of features must be processed efficiently.

### 4.3 Ecological and methodological implications

From an ecological perspective, the observed spectral responses reflect key plant physiological mechanisms under grazing disturbance (Xiang et al., 2025a, b). Moderate grazing stimulates compensatory growth and chlorophyll regeneration, leading to increased red-edge reflectance, whereas severe grazing reduces leaf area index (LAI) and internal scattering, lowering NIR reflectance. Concurrently, increased soil exposure elevates visible-band reflectance, generating a nonlinear response in the composite spectral signal. This “hybrid soil–vegetation effect” emphasizes that spectral variability in sandy grasslands arises from coupled changes in vegetation physiology, community composition, and soil optical properties.

Methodologically, this study demonstrates that multi-source UAV imagery combined with AIFS enhances classification performance while reducing redundancy, outperforming both full-feature and single-source approaches. The optimized feature set (idx9, idx8, idx7, NDVI, idx2, idx3, NDRE) achieved the highest classification accuracy and robustness, validating the efficiency of automated, data-driven feature selection in heterogeneous environments.

### 4.4 Limitations and future directions

Despite these advances, several limitations remain. The study was conducted on a controlled grazing trial with limited spatial coverage, which may not fully capture the spatial heterogeneity of sandy grasslands. In particular, the relatively small study area restricts the ability to test the generalizability of the proposed method across broader ecological gradients. The use of single-period UAV imagery restricted the analysis of seasonal dynamics. Additionally, some confusion persisted between light and moderate grazing levels, suggesting the need for features more sensitive to early degradation signals. Furthermore, although UAV-based data acquisition reduces many operational uncertainties, potential sources of error remain, including illumination variability, minor fluctuations in flight altitude, and atmospheric effects. In this study, flights were conducted under clear-sky conditions near solar noon to minimize illumination differences, and radiometric calibration panels along with the UAV’s RTK module were used to ensure consistent reflectance retrieval and high positioning accuracy. These measures help reduce uncertainty, but residual variability may still influence spectral responses.

Future studies should expand monitoring to diverse soil–vegetation types, extend the research area to larger and more heterogeneous sandy grassland regions, incorporate multi-temporal UAV datasets for dynamic assessments. Integrating medium- and high-resolution satellite data (e.g., Sentinel-2, GF-6) with UAV observations would also allow multi-scale validation and facilitate the application of the method to regional monitoring. Moreover, integrating structural information from LiDAR or radar could enhance discrimination under low vegetation cover. Further development of hybrid feature selection and deep learning approaches could also improve model robustness and generalizability.

## 5 Conclusions

This study utilized the fusion of visible and multispectral UAV data to systematically analyze spectral response features across different grazing intensities in sandy grasslands, which are characterized by low vegetation cover and substantial soil background interference. Integration of feature construction with an AIFS method improved the accuracy of grazing intensity classification. The main findings of this study are summarized as follows:

1. Visible and multispectral bands responded differently to grazing disturbance. Notably, the red edge and near-infrared bands were particularly effective in distinguishing between medium- and severe-grazing intensities. Nonlinear variations resulting from soil-vegetation interactions highlight the distinctive spectral characteristics of sandy grasslands.
2. Traditional vegetation indices (NDVI, GNDVI) were sensitive primarily to extreme grazing levels, whereas the proposed indices (idx7, idx9, idx14) outperformed them in distinguishing moderate grazing intensities, demonstrating strong regional adaptability.
3. The AIFS method efficiently reduced redundant information and enhanced model robustness, achieving the highest accuracy with the SVM classifier (OA=92.13%, Kappa=88.99%), surpassing the performance obtained using all features or single-source data.

The study demonstrates that multi-source UAV remote sensing, combined with intelligent feature selection, provides a promising approach for high-precision monitoring of grazing intensity in sandy grasslands, offering valuable insights for assessing grassland degradation and informing ecological management. However, the study is limited by the use of single-period data and small plot sizes. Future studies could incorporate multi-temporal and multi-scale observations, along with the integration of structural data, to further elucidate the dynamic processes and mechanisms underlying grazing disturbance. This study not only provides technical support for monitoring grazing intensity in sandy grasslands but also offers novel insights for ecological remote sensing research in low-vegetation-cover grasslands.

## Data Availability

The original contributions presented in the study are included in the article/supplementary material. Further inquiries can be directed to the corresponding author/s.
