# Correction: Integrating UAV visible and multispectral imagery to assess grazing-induced vegetation responses in sandy grasslands

**DOI:** 10.3389/fpls.2025.1774249

**Published:** 2026-01-16

**Authors:** Qiang Guan, Mingyang Jiang, Wen Du, Xueyan Chen, Baolong Yan

**Affiliations:** 1 College of Computer Science and Technology, Inner Mongolia Minzu University, Tongliao, China; 2 College of Information and Electrical Engineering, Shenyang Agricultural University, Shenyang, China; 3 College of Engineering, Inner Mongolia Minzu University, Tongliao, China; 4 Innovation Center for Intelligent Forage Equipment, Inner Mongolia Minzu University, Tongliao, China; 5 College of Grassland Science, Inner Mongolia Minzu University, Tongliao, China

**Keywords:** sandy grasslands, multi-source remote sensing, unmanned aerial vehicle, grazing intensity, spectral indices, feature selection

An incorrect grant number was provided for the Science and Technology Plan Project of Inner Mongolia Autonomous Region. The grant number was incorrectly listed as “2025SYFDZ0411”. The correct grant number is “2025YFDZ0025”.

The original version of this article has been updated.

